# Clival Ectopic Pituitary Adenoma Causing Cushing Syndrome

**DOI:** 10.1210/jcemcr/luad115

**Published:** 2023-09-25

**Authors:** Subramaniam Karthik, Tharun Krishna, Jai Richo Johnson, Jyothi Karikkanthra

**Affiliations:** Department of Endocrinology, Apollo Adlux Hospital, Angamaly, Kerala 683576, India; Department of Neurosurgery, Apollo Adlux Hospital, Angamaly, Kerala 683576, India; Department of Otorhinolaryngology, Apollo Adlux Hospital, Angamaly, Kerala 683576, India; Department of Pathology, Apollo Adlux Hospital, Angamaly, Kerala 683576, India

**Keywords:** ACTH dependent Cushing syndrome, Remission

## Abstract

The development of adenohypophysis by differentiating and detaching from the pharyngeal roof results in formation of a functional ectopic pituitary tissue around the Sella turcica. Of the ectopic sites in which pituitary adenoma occurs, clival adenomas are rare and the majority secrete prolactin. We report a case of ACTH-dependent Cushing syndrome. Magnetic resonance imaging revealed a clival mass with sphenoid sinus infiltration and ^68^Gallium (Ga) Dodecane tetra acetic acid—NaI3—octreotide positron emission tomography-computed tomography showed intense uptake in the region. Postoperative immunohistochemistry revealed ACTH and T-box Protein in T-cell positivity. With literature review, we found 5 reported cases of clival ectopic pituitary adenoma with Cushing syndrome. Clinical characteristics, imaging features, histology, and management of such masses have been discussed. In conclusion, ACTH-producing clival ectopic pituitary adenomas are rare, require differentiation from neuroendocrine tumors, and remit by multimodal therapy.

## Introduction

Cushing syndrome has remained 1 of the most difficult endocrine disorders to manage to date because localizing the source can be a challenge. Even in those patients with central localization, as confirmed by inferior petrosal sinus sampling (IPSS), ectopic location of a tumor around the sella turcica remains 1 of the reasons for disease persistence after surgery. In a review of 21 cases of ectopic ACTH-secreting pituitary adenoma in sphenoid sinus, preoperative diagnosis of ectopic pituitary adenomas (EPA) was not made in 9 (46%), and in 2 (10%) imaging did not show the ectopic location of the adenoma [1]. Hence, in a case of ACTH-dependent Cushing syndrome, suspecting an ectopic location of the tumor is of utmost importance when imaging shows a normal pituitary or when there is an empty sella.

EPA have been commonly reported in the sphenoid sinus, suprasellar region, clivus, cavernous sinus, and nasopharynx. A few uncommon locations include the superior orbital fissure, nasal cavity, maxillary sinus, and ethmoid sinus. Ectopic extracranial locations of the pituitary can be understood from embryology. Pituitary organogenesis is a complex, temporally, and spatially regulated event. The ascent and migration of the Rathke pouch to join with the neurohypophysis occurs when the Rathke pouch detaches from the pharyngeal roof. The spillover of cells during this process has been presumed to be the reason for ectopic pituitary tissue. Different lineages in adenohypophysis arise from difference in orientation of precursor cells in dorsal-ventral gradient of the paracrine fibroblast growth factor 8-bone morphogenetic protein 2 hormone milieu [2]. We describe a case of ectopic clival pituitary adenoma producing ACTH-dependent Cushing syndrome and review the literature.

## Case Presentation

A 56-year-old postmenopausal woman with no prior comorbidities was referred to us with uncontrolled diabetes mellitus, hypertension, persistent hypokalemia, and an infected left leg ulcer. Before being admitted, she had presented to another hospital, with altered sensorium and labored breathing and was diagnosed to have new-onset diabetic ketoacidosis associated with infected left leg scalds. She was managed in intensive care with an insulin infusion, broad-spectrum antibiotics, and severe hypokalemia requiring IV correction. Infected scalds evolved into abscesses and incision/drainage was performed (with the pus growing *Pseudomonas*). Because her glycemia, hypertension, hypokalemia, and sepsis worsened, she was referred to our hospital. On referral, she was on basal-bolus insulin, oral potassium supplementation, antihypertensives, and antibiotics.

On evaluation at our hospital, she had a hyperpigmented, flushed, and moon-like face; hypertrichosis over sideburns; atrophic skin over hands; and severe proximal myopathy of all 4 limbs. There were 2 purulent ulcers with necrotizing fasciitis in lower part of her left leg. Her biochemical reports are shown in Table 1. She had severe hypokalemia for which IV potassium replacement supplemented with spironolactone 100 mg/d was initiated. Dysglycemia was controlled with an insulin infusion and subsequently a basal-bolus insulin regimen; leg ulcers were managed with piperacillin-tazobactam infusion and debridement.

**Table 1.** Laboratory characteristics of the patient

Parameter	Patient's value	Normal value
HbA1c, % (mmol/mol)	8.7 (72)	<5.7 (<39)
Sodium, mEq/L	143	135-145
Potassium, mEq/L	2.2	3.5-4.8
pH in ABG	7.51	7.38-7.42
Bicarbonate, mmol/L	38	22-26
Total bilirubin, mg/dL (µmol/L)	1.4 (24)	<1.2 (<20)
ALT, U/L	50	<40
Alkaline phosphatase, U/L	352	60-300
Gamma glutaryl transpeptidase, U/L	104	<70
Hemoglobin, g/dL	9.8	13-15
ODST, mcg/dL (nmol/L)	28 (772)	<1.8 (<50)
Late night salivary cortisol, mcg/L (nmol/L)	37 (102)	<2 (<5.5)
ACTH, pg/mL (pmol/L)	126 (27)	<42 (<9.2)

Abbreviations: ABG, arterial blood gas; ALT, alanine transaminase; HbA1c, glycated hemoglobin; ODST, overnight dexamethasone test with 1 mg dexamethasone.

## Diagnostic Assessment

**Figure 1.**
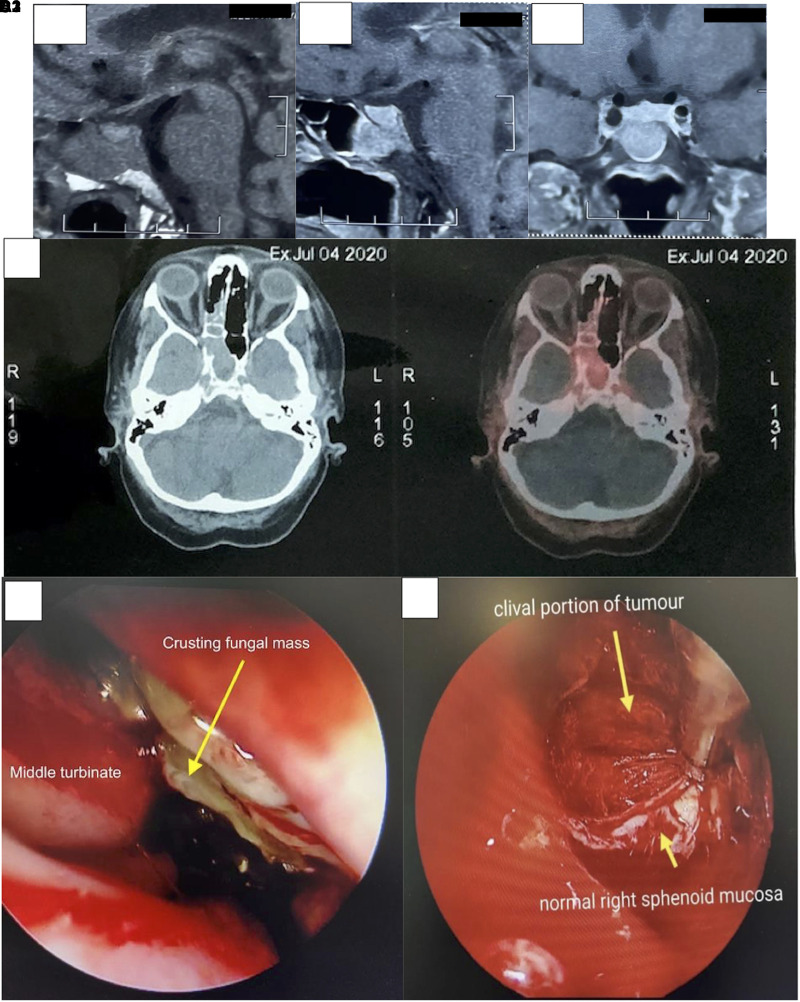
(A1) T1W MR of sella showing a normal sellar pituitary and heterogenous clival and right sphenoidal mass measuring 3 × 1.6 × 2 cm eroding the floor of sella. (A2, A3) Postcontrast sagittal and coronal cuts, respectively. (B) ^68^Ga DOTANOC PET-CT showing tracer-avid lesion in the right sphenoid sinus. Standardized uptake value maximum of the lesion was 4.7. (C) Incidentally detected crusting and eroding mass in the right nose, which was debrided and later turned out to be *Rhizomucor* spp. (D) Sphenoid sinus after debulking of the mass showing vascular clival tumor. Left sinus mucosa was seen as healthy tissue.

Because her biochemical reports confirmed ACTH-dependent Cushing syndrome, magnetic resonance imaging of the brain was done and showed a 3 × 1.6 × 2-cm mass with altered signal intensity in the basisphenoid of clivus, extending into the right sphenoid sinus and superiorly to the sella, eroding its floor (Fig. 1A). In T1-weighted (T1W) images, the normal pituitary gland was hyperintense to the sphenoid mass. Because IPSS would have resulted in a central gradient for both Cushing disease and clivus-sphenoid sinus ectopic ACTH-secreting tumor, we did functional imaging. Fluorodeoxyglucose positron emission tomography (FDG-PET) was nonrevealing but ^68^Gallium (Ga) Dodecane tetraacetic acid—NaI3—octreotide (DOTANOC) PET computed tomography (CT), showed somatostatin avidity in the sphenoid sinus mass (Fig. 1B). The standardized uptake value maximum of the sphenoid mass was 4.7, whereas that of the normal pituitary was 3.2.

## Treatment

To improve the metabolic complications, ketoconazole was started at a dose of 200 mg twice daily with monitoring of liver functions. After 1 week of initiation, she developed cholestatic liver injury and ketoconazole was stopped. Three weeks later, after normalization of liver enzymes, she underwent surgery. While undergoing transsphenoidal surgery, incidentally, a crusting soft-tissue mass was seen eroding the nasal septum and was debrided completely (Fig. 1C). The sphenoid tumor was debulked until the clival portion, which was highly vascular. The sellar dura was intact and the floor was dehiscent (Fig. 1D).

**Figure 2.**
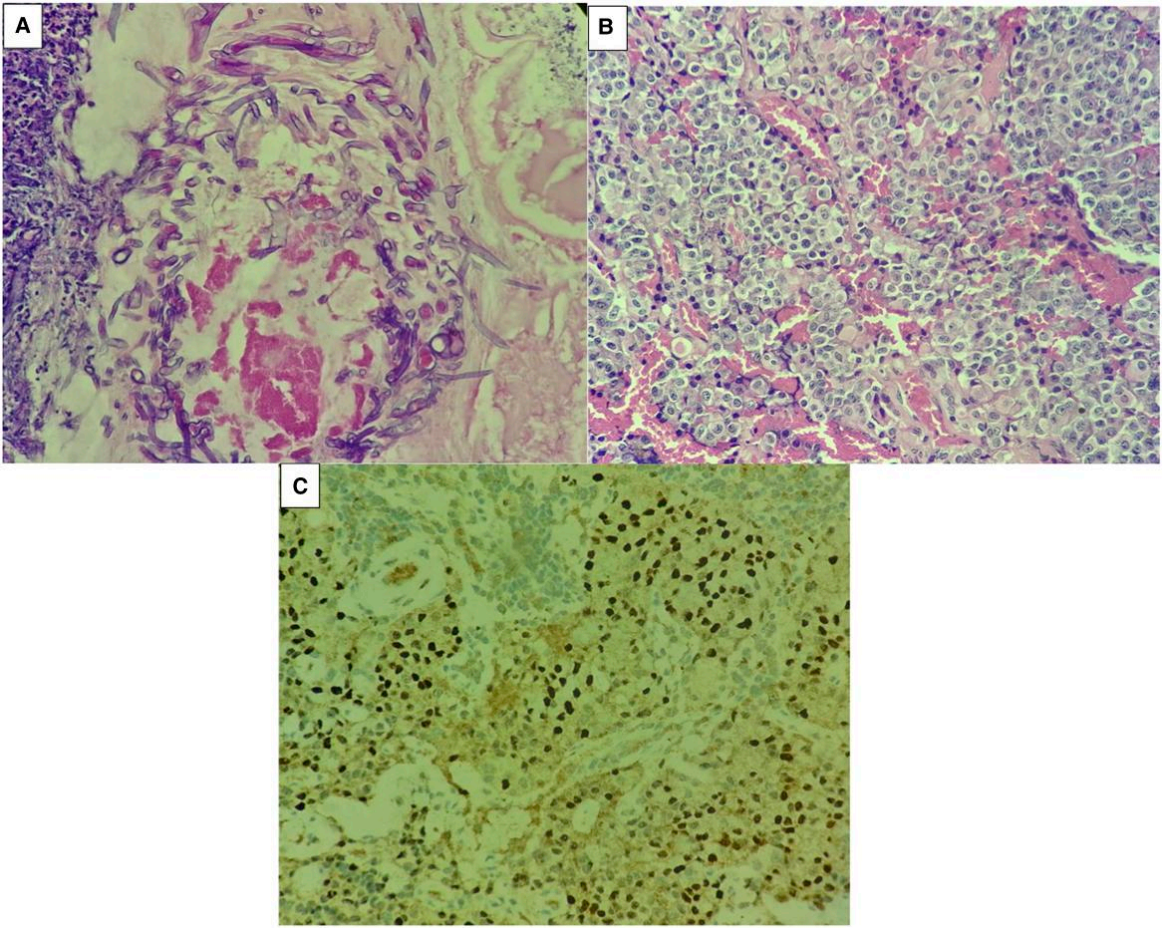
(A) Hematoxylin and eosin staining revealing broad, nonseptate hyphae from a nasal crusting mass showing necrotizing vasculitis and vessel wall infiltration with fungal hyphae. (B) Sphenoid mass histopathology showing a solid sheet of neoplastic cells with vascular stroma. Few cells show a clear cytoplasm, whereas others show eosinophilic clear cytoplasm. Crooke cell changes are not seen. (C) Immunohistochemistry showing TPIT-positive cells.

Histopathology and immunohistochemistry images are displayed (Fig. 2). The nasal crusting mass on hematoxylin and eosin staining was suggestive of *Rhizomucor* spp. ACTH immunohistochemistry was positive. Because neuroendocrine tumors and ectopic pituitary adenoma both have similar histology, can cause Cushing syndrome, and express neuroendocrine markers, to differentiate between them, we performed a pituitary-specific transcription factor T-box Protein in T cells (TPIT) immunohistochemistry, which was positive. Ki-67 index of the tumor was 2% to 3%.

## Outcome and Follow-up

The postoperative period was uneventful but there was no remission of Cushing syndrome (day 5 cortisol was 22 mcg/dL [607 nmol/L]). However, her insulin requirement, hypertensive medications, and potassium replacement reduced in postoperative period. She was started on IV liposomal amphotericin B in view of mucormycosis invading the nasal septum. Repeat nasal endoscopy after 1 week showed complete resolution of the lesion; hence, amphotericin was stopped after 10 days. After vitamin D supplementation, IV zolendronic acid was administered for osteoporosis.

Repeat magnetic resonance scans after 1 month revealed reduction in the size of the tumor by 50%, with a residual lesion in the clival region. Her morning cortisol was 16 mcg/dL (441 nmol/L) and there was further decrement in diabetic and hypertensive medications. She underwent stereotactic radiosurgery for the residual clival mass 3 months after surgery. Six months after radiotherapy, her salivary cortisol was 4 mcg/L (11 nmol/L). Her diabetes and hypertension were well controlled with a single medication each. At the time of reporting—3 years after surgery—she has been keeping well without any further symptoms.

## Discussion

Clival masses have a wide differential diagnosis and include chordoma, chondrosarcoma, plasmacytoma, metastasis, osteoblastoma, meningioma, developmental cysts, and EPA. EPA around sella are very rare and about 180 cases have been reported to date [3]. In an extended review of the literature, using terms “clivus,” “ectopic,” “pituitary adenoma,” “ACTH producing,” “extra-pituitary,” and “Cushing syndrome” in PubMed and Web of Science, we were able to identify 5 previous published reports of ACTH-producing EPA arising from the clivus with or without extension into surrounding tissue(s) (Table 2) [4‐8]. A recent comprehensive review of EPA showed that although sphenoid sinus adenomas never infiltrated the clivus, 40% of clival adenomas extended into the sphenoid sinus [3]. Moreover, clival, nasopharyngeal, and sphenoid EPA were more likely to preferentially secrete prolactin, TSH, and ACTH, respectively. Because clival EPA causing Cushing syndrome is very rare and this is the sixth report of such a tumor, we present a comprehensive literature review regarding all 6 cases.

**Table 2.** Reported cases of ACTH-producing ectopic pituitary adenomas in the clivus

No.	Age/sex	Symptoms	Characteristics of the mass	IPSS	Histopathology	Immunohistochemistry markers	Postoperative status	Follow-up duration	Reference
1	15/F	Cushingoid features, headache, diplopia, facial numbness	Right extrasellar extending from clivus to superior orbital fissure	No	Chromophobe adenoma	NM	Received radiotherapy. In remission	NM	4
2	58/F	Nasal obstruction, anosmia, blurred vision	3 × 3-cm midline mass in nasopharynx and clivus	No	Chromophobe adenoma	ACTH positive, GH/PRL negative	Received EBRT. In radiological remission	1 y	5
3	60/F	Sudden-onset headache, uncontrolled diabetes, hypertension	5.5 × 4.5 × 3-cm mass destroying clivus, extending to sphenoid sinus and middle cranial fossa	No	Chromophobe adenoma	ACTH positive, GH/PRL/FSH/LH/CRH negative	Biochemical/radiological remission	3 y	6
4	20/F	Failed transsphenoidal surgery for Cushing syndrome	Medial wall of right cavernous sinus and right upper of clivus	Central gradient	Chromophobe adenoma- encapsulated	ACTH positive	Clinical/biochemical remission	18 mo	7
5	53/F	Resistant hypertension, bone fractures, cushingoid features	3 × 4.5-cm clival mass extending to sphenoid sinus	Central		CK/SYN/CgA/ACTH positive GH/LH/TSH/PRL negative	Partial remission	10 d	8
6	56/F	Cushingoid features, new-onset diabetes, hypertension	3 × 1.6 × 2-cm mass involving basisphenoid and sphenoid sinus	No	Chromophobe adenoma	ACTH/LMWCK/TPIT positive, TSH/LH/FSH/GH/PRL negative	Underwent stereotactic radiosurgery/clinical remission	3 y	Current case

Abbreviations: CgA, chromogranin A; CK, cytokeratin; CRH, corticotropin-releasing hormone; EBRT, external beam radiotherapy; IPSS, inferior petrosal sinus sampling; LMWCK, low-molecular-weight cytokeratin; NM, not mentioned; PRL, prolactin; SYN, synaptophysin; TPIT, T-box Protein in T cells.

All of the cases reported were in women. The presenting symptoms were predominantly classical Cushing syndrome phenotype except in 1 case (#2), in which it was purely mass related (nasal obstruction and headache). Except for 1 patient (#4), all others had a macroadenoma with infiltration into surrounding structures.

It has been hypothesized that the ectopic pituitary tissue arises because of failure of migration of pituitary tissue during embryonal period and, in that case, empty sella might be noted [3]. None of the 5 cases had empty sella. In a review of the literature, of 149 patients with EPA undergoing magnetic resonance imaging, ∼95% were iso-hypointense in T1W imaging and ∼90% were iso-hyperintense in T2-weighted imaging [3]. In this cohort of clival tumors, T1W images were hyperintense in 4 cases (#2, #3, #5, and #6) and hypointense in 1 patient (#4).

An important investigation in ACTH-dependent Cushing syndrome with normal sellar imaging is IPSS. In a review of literature of ACTH-producing EPA, 100%, 83%, and 79% of suprasellar, sphenoid sinus, and cavernous sinus tumors had a central gradient, respectively. Caution needs to be exercised while interpreting IPSS with a central gradient in those having normal/empty sella because of shared venous drainage between ectopic pituitary tissues and the pituitary gland. In cases in which there are obvious radiological lesions around the sella, one cannot be sure of the source of Cushing syndrome (whether ACTH is from pituitary vs EPA). In case of occult radiological lesions around the sella (as was the case in patient #4), with IPSS showing central gradient, sellar exploration and surgery should be undertaken.

Normal pituitary expresses somatostatin receptors (SSTR) 1, 2, and 5. Novel somatostatin analogs used in nuclear imaging binds to avidly to SSTR 2 and SSTR 5 (which are usually overexpressed in neuroendocrine tumors). The role of functional nuclear imaging in the diagnosis of ACTH-secreting EPA has been evaluated in a few patients. One was a case of ^8^F-FDG and ^8^F-DOPA PET-CT negative but ^68^Ga-DOTATATE PET-CT positive tumor in the right sphenoidal sinus [9] and the second was an ^18^F-Choline PET-CT localized left maxillary sinus tumor in a patient in which initial ^18^F-FDG and ^68^Ga-DOTANOC scans were negative [10]. In our patient, even though ^18^F-FDG was negative, ^68^Ga-DOTANOC localized the lesion and the lesion had higher standardized uptake value maximum compared with normal pituitary tissue.

The closest differential for ACTH-producing EPA is carcinoid tumor in the head and neck region. Though rare, sphenoid sinus carcinoid tumors have been reported in the literature. The histopathological features overlap between the 2 in the form of differential patterns of growth within the tumor, mild to moderate fibrovascular stroma, small dense cells with nuclear crowding, and overlapping. Destruction of nasal epithelium, numerous mitoses, smudging, and crush artifact, if present, suggest a neuroendocrine tumor. Imaging characteristics do not differentiate the 2. Neuroendocrine markers may be expressed in both tumors—about 97%, 76%, and 71% of EPA express synaptophysin, neuron-specific enolase, and chromogranin. Pituitary-specific transcription factors (TPIT, PIT-1, SF-1) are unique and specific to EPA.

Surgical outcomes have been reported in ACTH-secreting EPA. Remission rates were 94%, 76%, and 60% in sphenoid sinus, suprasellar, and cavernous sinus tumors respectively. Two patients (#3 and #4) underwent a second surgery for complete resection of a clival tumor and were in remission after the second surgery. Another 3 patients underwent extensive debridement first and required postoperative radiotherapy. Although cases #1 and #2 received external beam radiotherapy, our patient received stereotactic radiosurgery. The long-term follow-up was not available in cases #1 and #5 but the other 3 cases were in remission.

In conclusion, we summarize the findings of ACTH-producing clival EPA in this paper. Testing for the pituitary hormone hyperfunction is of utmost importance in working up a clival mass and, in any case of doubt, immunohistochemistry must be done for accurate diagnosis. Because of bony involvement, extensive surgery and added radiotherapy might be required in these cases.

## Learning Points

- Ectopic pituitary tissue by itself is rare and such tissue becoming adenomatous and functional is much more uncommon.
- Parasellar adenomas that secrete ACTH may show central gradient in inferior petrosal sinus sampling.
- Multimodal therapy requiring surgical debulking and stereotactic radiotherapy with or without medications to control cortisol excess remains the cornerstone of case management.

## Contributors

S.K. was involved in diagnosis and evaluation. T.K. and J.R.J. were involved in surgical and postoperative management. J.K. reported histopathology and did immunohistochemistry. All authors were involved in drafting and revising the manuscript.

## Data Availability

Data sharing is not applicable to this article as no datasets were generated or analyzed during the current study.

## Abbreviations

CT: computed tomography
DOTANOC: dodecane tetraacetic acid—NaI3—octreotide
EPA: ectopic pituitary adenoma
FDG: fluorodeoxyglucose
IPSS: inferior petrosal sinus sampling
PET: positron emission tomography
SSTR: somatostatin receptor
T1W: T1-weighted
TPIT: T-box Protein in T cells
